# Generalized music therapy to reduce neuroactive drug needs in critically ill patients. Study protocol for a randomized trial

**DOI:** 10.1186/s13063-024-08220-8

**Published:** 2024-06-12

**Authors:** Giovanni Mistraletti, Anna Solinas, Silvia Del Negro, Carlotta Moreschi, Stefano Terzoni, Paolo Ferrara, Katerina Negri, Davide Calabretta, Paolo Formenti, Angelo Formenti, Michele Umbrello

**Affiliations:** 1 Dipartimento Di Fisiopatologia Medico-Chirurgica E Dei Trapianti, Università Degli Studi Di Milano, Milan, Italy; 2 SC Rianimazione e Anestesia, Ospedale Civile di Legnano, ASST Ovest Milanese, Milan, Italy; 3 Dipartimento Di Salute Mentale, AUSL Piacenza, Piacenza, Italy; 4 Dipartimento Di Scienze Della Salute, Università Degli Studi Di Milano, Milan, Italy; 5 Servizio Di Psicologia Clinica, Ospedale San Paolo – Polo Universitario, ASST Santi Paolo E Carlo, Milan, Italy; 6 SC Anestesia, Rianimazione e Terapia Intensiva; ASST Nord Milano, Ospedale Bassini, Cinisello Balsamo, Italy; 7 Centro Sperimentale Regionale Della Voce E Della Deglutizione “E. De Amicis”, Milan, Italy

**Keywords:** Music therapy, Critical illness, Intensive care units, Conscious sedation, Psychological stress

## Abstract

**Background:**

Critically ill patients are exposed to several physical and emotional stressors, needing analgesic and sedative drugs to tolerate invasive procedures and the harsh intensive care unit (ICU) environment. However, this pharmacological therapy presents several side effects: guidelines suggest using a light sedation target, keeping critically ill patients calm, conscious, and cooperative. Personalized music therapy (MT) can reduce stress and anxiety, decreasing the need for drugs. The aim of the current investigation is to compare different approaches for MT in the ICU: a personalized approach, with music selected by patients/families and listened through headphones, or a generalized approach, with ambient music chosen by a music therapist and transmitted through speakers. Primary outcome: number of days “free from neuroactive drugs” in the first 28 days after ICU admission. Secondary outcomes: total amount of neuroactive drugs (midazolam, propofol, morphine, fentanyl, haloperidol), stress during ICU stay (sleep at night, anxiety and agitation, use of physical restraints, stressors evaluated at discharge), the feasibility of generalized MT (interruptions requested by staff members and patients/families).

**Methods:**

Randomized, controlled trial with three groups of critically ill adults: a control group, without MT; a personalized MT group, with music for at least 2 h per day; a generalized MT group, with music for 12.5 h/day, subdivided into fifteen 50-min periods.

**Discussion:**

One hundred fifty-three patients are expected to be enrolled. This publication presents the rationale and the study methods, particularly the strategies used to build the generalized MT playlist. From a preliminary analysis, generalized MT seems feasible in the ICU and is positively received by staff members, critically ill patients, and families.

**Trial registration:**

ClinicalTrials.gov Identifier: NCT03280329. September 12, 2017.

**Supplementary Information:**

The online version contains supplementary material available at 10.1186/s13063-024-08220-8.

## Introduction

### Background and rationale {6a}

Treatment in an intensive care unit (ICU) is a significant cause of physical stress and emotional difficulties for patients and their families [1]. Anxiety and agitation are frequent among critical patients; they can be caused by organic and metabolic alterations (hypoxemia, hypercapnia, acidosis, hypoglycemia, hypo/hypernatremia, sepsis, hypovolemia, alcohol, abstinence,…), which must be examined and treated first. Moreover, the harsh ICU environment, full of advanced technological devices, constitutes an unfamiliar space for the patient, being a potential cause of fear and panic. The leading causes of stress in the critically ill are the lifesaving treatments such as mechanical ventilation (MV) or invasive procedures, movement restriction, pain and the inability to express it verbally, the light and sound in the environment, the loss of interaction with loved ones, and sleep deprivation [2].

Anxiety management involves several interventions, including first limiting stressors, with verbal reassurance and environmental measures, and avoiding analgesics, sedatives, and antipsychotics. The use of these drugs is fundamental in critically ill patients; however, it should be avoided as far as possible, as it has been recognized as being related to significant side effects like prolongation of MV, increased length of stay [3, 4], muscle fatigue, asthenia, delirium, post-traumatic stress disorder [5], hemodynamic impairment, cardiac rhythm disturbances [6, 7], sepsis [8], ileum, and an increased risk of ventilation-associated pneumonia.

Despite using tools to evaluate pain, agitation, and delirium and protocols for their management, many patients suffer frequent anxiety, discomfort, distress, hallucination, or disorientation. Inadequate treatment of these conditions is associated with increased sympathetic activity, which causes hypertension, tachycardia, dyspnea, and increased myocardial oxygen consumption [9]. There is, therefore, a clear need for new strategies and instruments to improve the comfort of critically ill patients by relieving pain, anxiety, stress, and agitation and minimizing the need for neuroactive therapies.

Among non-pharmacological treatments, sleep-inducing relaxation techniques, communication with the patient, music therapy (MT), and physical support have proved effective [10–12]. The use of music as a complement to treatment has long been described in the literature [13]. MT represents a complementary and integrative therapeutic approach that harnesses the use of music to promote the well-being and healing of patients with a wide range of clinical conditions, being able to offer significant benefits in the critically ill patient having no contraindications and being easily applied at the bedside [14, 15]. MT may reduce delirium [16], anxiety [17], and agitation [18] in critically ill patients; by facilitating relaxation [19], it improves cardiovascular parameters [20, 21] and dampens biological stress [22]. These findings have solid neurological [23] and hormonal [24] bases.

Usually, MT is based on the patient’s preferences, recorded by a music therapist to identify the appropriate tracks by talking to patients themselves or their families [25]. However, several music-based interventions have been proposed in the literature, considering distinct music selectors, different delivering methods (headphones vs. ambient music), and different treatment protocols based on duration, number, and frequency of sessions and music genre [26–29].

Despite their apparent usefulness, these techniques are difficult to standardize and the development of easily generalizable and applicable protocols could further benefit their application.

### Objectives {7}

The research question that drove the study’s design here described is to investigate the effectiveness of MT in reducing the need for analgesics and sedatives and relieving anxiety while re-establishing standard sleep patterns. Our main outcome is the decrease of the dosage of all the neuroactive drugs administered during the ICU stay. This study compares two approaches for the music: personalized, where patients select the tracks and listen through headphones, and generalized, with playlists transmitted through speakers to the room.

## Methods

### Trial design, setting, and authorization {8} {9}

This is a randomized, controlled, single-center, open-label trial carried out in the ICU of the San Paolo General Hospital in Milan, Italy, which comprises three separate rooms with two or three beds each. The Ethics Committee of Milan Area 1 (Prot. 9827 of April 13, 2017) authorized the trial.

### Outcomes {12}

The main hypothesis is that both personalized and generalized MT can significantly reduce the need for neuroactive drugs in critically ill patients. We will investigate whether MT increases the number of days “free from neuroactive therapy” (analgesics, sedatives, anxiolytics, and antipsychotics) [30] in the first 28 days after ICU admission and, as secondary outcomes, whether it reduces the total of ten commonly used sedative and analgesic ICU medications (midazolam, lorazepam, propofol, dexmedetomidine, morphine, fentanyl, remifentanil, hydroxyzine, haloperidol, quetiapine). Sedative use will be measured as the daily sedative drug intensity score and dose frequency. The reduction of ICU stress will be measured using the validated ICU Environmental Stressor Scale (ICUESS) scale, which rates the perception of stressors during the whole ICU stay [31].

### Consent {26a}

Written informed consent has been gathered by the investigators from all competent patients; for temporarily non-competent ones, permission to use the personal data already collected has been granted as soon as they become competent, according to the ethics committee’s indications.

### Additional consent provisions for collection and use of participant data and biological specimen {26b}

Not applicable.

### Eligibility criteria {10}

All critically ill patients were screened for enrolment as they are admitted to the ICU, according to these inclusion criteria: age > 18 years, expected mechanical ventilation > 48 h. The exclusion criteria are as follows: expected Glasgow Coma Scale < 12 at ICU discharge, hearing loss, advanced neurodegenerative dementia, any unbalanced psychiatric condition in their history, a change of bed or room during the ICU stay.

### Interventions and blinding {11a} {6b} {11b} {11c} {11d} {16a} {16b} {16c}

**Fig. 1:**
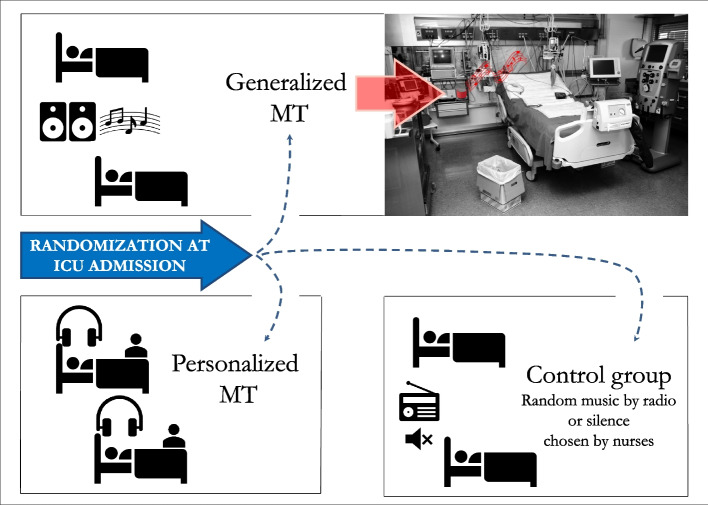
Group allocation at ICU admission

Participants were randomly allocated to three groups, with a parallel assignment at ICU admission, applying an electronic minimization algorithm. Depending on their assigned group, they were admitted to a specific ICU room by the staff intensivist in charge at the time of ICU admission (Fig. 1).

In the control group, patients could hear the background sounds (alerts, voices) of the ICU environment; listening to unspecified radio programs is allowed, according to medical/nursing judgments, as per usual local practice. Patients in the personalized MT group could listen to music chosen according to their preferences, for all time they wanted, with a minimum of 2 h per day. MT was selected from specific 30-min playlists of all the music available. They can ask for other particular tracks or types of music, if not immediately available. For comatose or deeply sedated patients, music was chosen by their relatives after an interview on the patient’s presumed preferences. The meeting with the music therapist was carried out within 24 h of randomization, and the Music Assessment Tool (MAT) was used to collect patients’ preferences, reported in the supplementary materials.

**Fig. 2:**
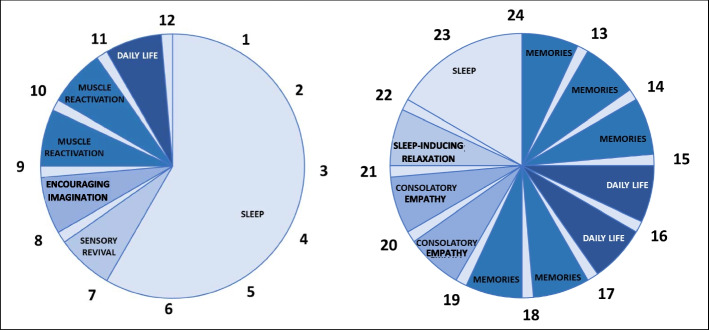
Generalized music therapy during the clock hours. The tracks composing the playlist for the generalized MT were chosen according to the purposes shown. Every hour, there were 50 min of uninterrupted music and 10 min of silence to avoid habituation effect. From 22 to 7, the silence was used to promote physiological sleep. MT, music therapy

Patients allocated to the generalized MT group were admitted to a room where music was broadcast for 12.5 h per day, based on a specific “weekly playlist,” as follows. Sound was on from 07:00 to 22:00 (clock hours, see Fig. 2), with a 10-min break every 50 min of music, and silence at night, from 22:00 until 07:00 in the subsequent morning. Music was transmitted through the environment with specifically designed speakers at a controlled volume (5 dB above the ICU noise). Tracks were from both classical, modern, and so-called contemporary music (considering commercial pieces composed since the mid-twentieth century), chosen for easy listening, and selected according to the hours per day needed to restore circadian rhythm and predictable activities of care (hygiene, food, other therapies, physiotherapy, visits from relatives, etc.). Tracks were mixed to ensure continuity and fluidity of listening. The selection of music in the generalized group occurred after the music therapist had conducted a week-long participant observation period at the center’s ICU. During this observation period, the therapist was able to analyze the different phases of life on the ward to the point of defining the criteria for the choice of tracks shown in Table 1.

**Table 1:** Purposes of the time slots in generalized MT

Slot	Time	Aim	Action taken
1	07:00–08:00	Sensory revival	Gradual, gentle waking of the senses starting from hearing. Evoking luminous visual images, caresses, or light tactile emotions. Inducing feelings of welcome and serenity. Progressive acceleration of heart rate and increasing brain activity
2	08:00–09:00	Encouraging imagination	Encouraging recollection of various life scenes, stimulating the imagination, opening up, and enriching the real visual horizon, which is inevitably limited in hospital
3	09:00–11:00	Muscle reactivation	Accompanying physiotherapy and encouraging gentle, slow but articulated movement, with deep, relaxed breathing. Lightening the fatigue and pain of physical mobilization
4	11:00–12:00	Daily life	Transmitting positive emotions (joy, serenity, vivacity, tenacity, strength, courage, etc.). Keeping the patient awake by synchronization of a medium–high heart rate, activation of cognitive functions of attention, concentration, and judgment
5	12:00–15:00	Memories	Making patients feel someone is caring for them, not just as a “body” but as a “person,” by arousing pleasant memories, sensations, feelings, and emotions
6	15:00–17:00	Daily life	Transmitting positive emotions (joy, serenity, vivacity, tenacity, strength, courage, etc.). Keeping the patient awake by synchronization of a medium–high heart rate, activation of cognitive functions of attention, concentration, and judgment
7	17:00–19:00	Memories	Making patients feel someone is caring for them, not just as a “body” but as a “person,” by arousing pleasant memories, sensations, feelings, and emotions
8	19:00–21:00	Consolatory empathy	Tuning in to the patients’ suffering so they “come back into themselves,” making them feel justified and understanding their pain or suffering while at the same time transmitting hope. Making them feel accompanied and cared for, on the path to recovery. Slowing the heart rate to encourage relaxation
9	21:00–22:00	Sleep-inducing relaxation	Neutralizing all the emotions, sensations, images, and motor stresses of the day, to induce relaxation, tranquility, and sleep

Between 07:00 and 22:00, seven playlists for the generalized MT were prepared by the music therapist, following the shared criteria listed here, in order to drive the patients’ daily activities, shepherd their thoughts, and facilitate restoration of physiological sleep-waking cycles. From 22:00 to 07:00, the speakers were silent

### Provisions for post-trial care {30}

There was no anticipated harm and compensation for trial participation, and no provision for post-trial participation was planned in the protocol.

### Data collection and participant timeline {13} {18a} {19}

**Fig. 3:**
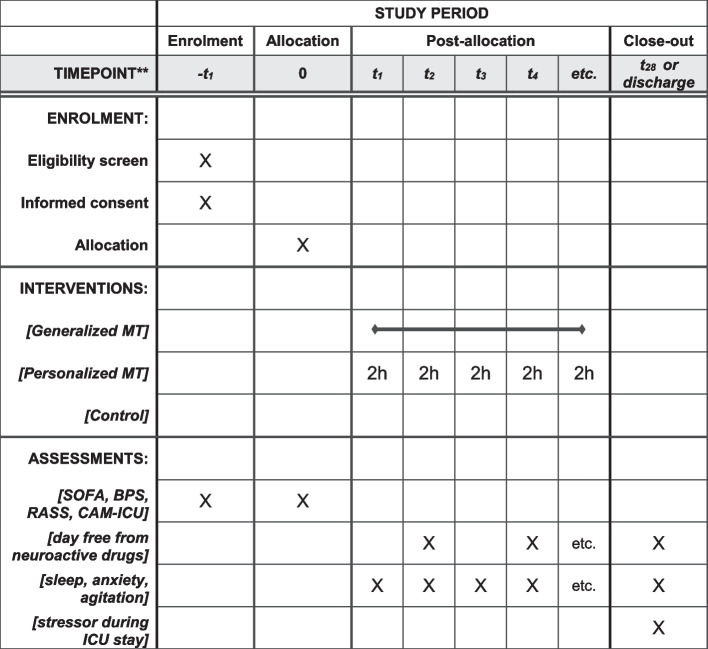
SPIRIT schedule of enrolment, interventions, and assessments. The figure depicts the overall schedule and time commitment for trial participants. MT, music therapy; SOFA, Sequential Organ Failure Assessment Score; BPS, Behavioral Pain Score; RASS, Richmond Agitation-Sedation Scale; CAM, Confusion Assessment Methods; ICU, intensive care unit

For each participant, the following information was recorded: where admitted from, the reason for hospitalization, comorbidities, the Simplified Acute Physiology Score 2 (SAPS II) after 24 h in the ICU [32], the daily Sequential Organ Failure Assessment (SOFA) score [33], and Glasgow Coma Scale (GCS) [34]. At each change of nursing shift, vital signs, doses of neuroactive drugs, Behavioral Pain Scale (BPS) [35], Richmond Agitation Sedation Scale (RASS) [36], Confusion Assessment Method for the ICU (CAM-ICU) [37], use of physical restraints, and incidence of adverse events were recorded. Figure 3 depicts the overall schedule and time commitment for trial participants.

In the room with generalized MT, the number of times the music was interrupted was recorded, while in the room with personalized MT, the total time the patients listened to music was recorded. On the day of ICU discharge, the Mini-Mental State Examination (MMSE) [38] was administered. If the score was higher than 24 points, the following tests were done: ICU Environmental Stressor Scale (ICUESS) [31], a Likert-type questionnaire to measure the stressfulness of commonly occurring items in the ICU, and the Short Screening Scale for Post-Traumatic Stress Symptoms (SSS-PTSS) [39], a seven-question survey to screen for the probability of a post-traumatic stress disorder.

Data were gathered daily, using values and drugs listed on the patient’s chart. Patients were assigned an ID to ensure their privacy. The ICUESS and SSS-PTSS questionnaires were recorded within 3 days from when the patient is discharged from the ICU. Finally, also, the hours of sleep have been recorded for each patient. All data collection sheets are available in Additional file 1 in the Italian version used.

### Plans to promote participant retention{18b}

Not applicable as all data were gathered during hospital stay.

### Plans for collection of biological specimens for genetic or molecular analysis {33}

Not applicable.

### Confidentiality {27}

All study-related information were stored securely at the study site. All participant information were stored in locked file cabinets in areas with limited access. All records that contain names or other personal identifiers, such as locator forms and informed consent forms, were stored separately from study records identified by code number. All local databases were secured with password-protected access systems.

### Personalized MT

ICU patients assigned to the personalized MT were screened with the Music Assessment Tool (MAT) [40] to determine their music preferences. The Italian version of MAT used is reported in Additional file 1. In case of neurological inability because of acute organic/metabolic disease or because of sedation, the MAT was completed with the help of the patients’ families. Then, the “preferred music” was transmitted for at least 2 h per day through a portable system and headphones, and patients could listen for the 2 h/per day until recovery of their neurological function. Neurologically competent patients could listen to their preferred music as long as they want.

The music therapist prepared several 30-min playlists; each patient (or his/her family) was invited to select at least four of these playlists—or to choose other genres. In this case, the music therapist prepared different playlists for the next day to meet these requests. If a patient preferred not to listen to music, s/he was not obliged, and his/her choice was recorded. Finally, also, the types of playlists and the time spent listening each day were recorded.

### Generalized MT

The music tracks on the playlist for the generalized MT were chosen by a music therapist according to the characteristics presented in Table 1. For example, in the early hours of the day, we offered songs that encouraged gentle awakening, passing gradually to imaginative stimulation and encouraging the body to wake. Towards the end of the day, the rhythm slowed to encourage patients to relax and accompany them toward sleep.

Other kinds of music were intended for the total daily ICU routines (therapy, mobilization of patients, relaxation, visits from families) and to reactivate emotions (to evoke recollections and motivate the patient for complete recovery) [41].

Seven different playlists, with the duration on 15 h, were drawn up for each day of the week. Different tracks were chosen according to these rules:

- Non-aggressive, simple, regular music (except for slots 4 and 6, where significantly more vigor is required)
- Genres as varied and popular as possible, excluding niche music such as Gregorian chant, Neapolitan melodies, free jazz (to name just some), heavy metal, hard rock, and techno/disco numbers
- Extreme variety of styles and ages. Exclusion of non-widely accepted or experimental styles
- Preferably high-quality recordings and performances
- Repertoire from Western culture, generally after the year 1600
- Each item is selected after analyzing the phono-symbolic significance of the music while bearing in mind that it is impossible to predict what kind of synesthetic associations may be aroused in each patient since they are unique and differ widely for everyone

The songs were selected by a music therapist with 30 years of experience, mainly working in the psychiatric field, and based on dozens of interviews with people of various ages.

Additional file 1 gives a complete description of the generalized MT methods. Besides the list of rules for selecting tracks, also presented are the criteria for each time slot and the technical features used in preparing the playlists. A comprehensive list of the Italian musicology books used by the music therapist is also available in Additional file 1.

### Statistical analysis and sample size calculation {14} {17a} {17b} {20a} {20b} {21b} {20c} {31c}

Given the nature of the study, blinding was not possible for treating physicians and for investigators who collected the data. Assuming the possibility of transforming the data with the Blom method in order to obtain normal distribution and therefore higher power with the same sample size, and taking an effect size of 0.30 and doubling of the primary outcome parameter (days without needing neuroactive drugs) in the two intervention groups compared to the control group, and between the two interventions, 53 subjects per group were required for a power of 80%. This sample was also sufficient for comparing repeated measurements on the same patient. For this, a linear model will be used, which requires 51 subjects per group, assuming a 0.5 correlation coefficient between the measures and an effect size of 0.25. The power of the sample was calculated with SAS software. No interim analyses are planned. Missing data will not be imputed. The full protocol, participant-level data, and statistical code will be held by the principal investigator.

### Steering committee, adverse events, and data monitoring committee {5d} {21a} {23} {21b} {22}

Given the single-center nature of the study, members of the steering committee were always available on site to provide day-to-day support for the study. The steering committee has been meeting monthly to discuss the progress of the project and provide feedback to the operators involved in data collection.

The ethics committee met only at the beginning of the project but remained at the disposal of the other investigators for the duration of the protocol in case of emergence of problematic issues.

No adverse events were expected to occur given the nature of the interventions and, for this reason, no data monitoring committee was needed.

### Plans for communicating important protocol amendments to relevant parties (e.g., trial participants, ethical committees) {25}

Any modifications to the protocol that could have an impact on the conduct of the study, or could have affected patient safety, including changes of study objectives, study design, patient population, sample sizes, and study procedures, would have required a formal amendment to the protocol to be approved by the ethics committee prior to implementation. However, no protocol amendments occurred during the recruitment period.

### Dissemination plans {31}

The study results will be released to the participating physicians, patients, and the general medical community.

### Preliminary qualitative data: the ICU staff members’ viewpoints

During the whole study, the comments of the healthcare staff working in the generalized MT milieu were collected. Seven weeks from the beginning of the study, physicians and nurses were asked if they found the music disturbing. They have reported that the music can be slightly distracting in the first few hours (“When we are doing some specific tasks, at the start of the *new hour of music*—especially if it’s known—may be distracting”) or in rare, specific conditions (“It would be better to turn off the speakers when we are doing tracheostomy or some other bedside intervention: the music, the sounds of the monitor, and us discussing things together … it’s chaotic!”). However, these comments have decreased over time, as being “immersed” in a musical atmosphere becomes a habit (“It’s nice to have these background songs, even though after a week I’ve already heard them all. It’s comforting to arrive and hear them” or “In the morning, during the nursing and attending to the patient’s hygiene, it’s very nice to have this kind of music: it would always be!”).

Many comments have been made on the choice of music—some deemed it inappropriate (“Christmas carols in the summer are silly…”) or it did not meet a patient’s musical tastes (“Hearing a rubbishy Italian song like *Azzurro* is not acceptable after the guitar solo of *Sultans of swing*: Mark Knopfler wouldn’t want it!”). Some have asked for more, different playlists (“To start with, it was a pleasant novelty, but after a while, the repetition of the tracks gets boring.”). These negative comments were reported by a minority of the staff since the wide variety of styles and tracks mainly satisfied everyone’s preferences (“I prefer to work in the generalized MT room instead of the control room because on the radio there’s so much more talk or advertising than good music.”).

Generalized music was considered better because it did not hinder clinical procedures and did not make the workload any heavier (“But isn’t it better to use loudspeakers for everyone? The headphones often come off when dressings are done, or during exercise.”). The headphones for personalized music can prove obstructive in some clinical maneuvers (e.g., mobilizing patients and physiotherapy), or they cannot be used because of particular clinical conditions (e.g., non-invasive mechanical ventilation with helmet CPAP or with some types of full-face mask, during an EEG, prone positioning, etc.).

### Expected results

Several studies have reported the positive effects of personalized music-based interventions [14, 16]; the present study aims to verify whether similar results can be obtained with generalized MT. The expected benefits for patients are an increase in sleep at night, reduction of anxiety and manifestations of psychomotor agitation, lower anxiety, and reduction in the frequency and duration of physical restraints.

**Fig. 4:**
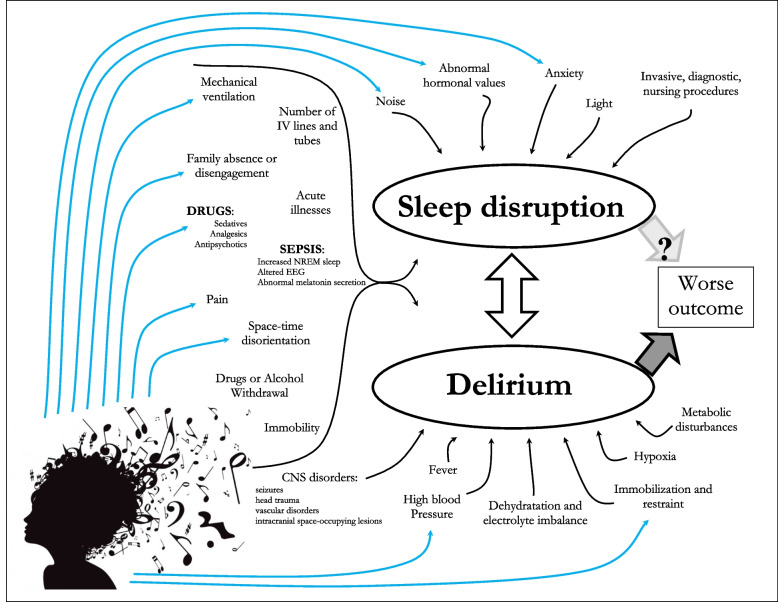
Potential targets of music therapy. Representation of the potential targets of music therapy in the complex interactions between sleep disruption and delirium with increasingly worse outcomes in critically ill patients

Sleep deprivation—in quality and quantity—is widespread in the ICU, as is delirium [42]; these pathological conditions have a range of causes (Fig. 4). We hardly expected that normalization of the sleep-waking rhythm in the generalized MT group guided by a sound-full day and a silent night, with particular attention to the waking and falling asleep phases, could lower the prevalence of delirium and consequently possibly reduce the need for neuroactive drugs. Data on sleep distribution during the morning-afternoon-night phases were collected.

## Discussion

If the expected results are achieved, they could offer an important advantage in the wellbeing of critical patients, both during their ICU stay and after discharge. Hopefully, MT could have significant implications regarding assistance, outcomes, and length of ICU stay.

If generalized and personalized MT give similar results, there could be various advantages. Equipping each bed with a music player and ensuring that nurses manage it may not be feasible due to the costs and additional workload. Moreover, it could be problematic to have to promptly upload onto a music player the wide range of musical genres that respond to the tastes of each patient. On the other hand, having a speaker near each bed to automatically spread the music in the ICU ward could be pleasant for patients, families, and staff members.

Another advantage of generalized MT might be the possibility of using these ICU playlists in other hospital wards too. The literature indicates that MT can reduce stress and improve work environments and perceived patient care [43].

### ICU humanization: building a healing environment

Critically ill patients experience various distressing symptoms while in the ICU, including pain, agitation, delirium, weakness, and sleep deprivation. Because of the complexity of caring for these patients, the symptoms are frequently managed by keeping them heavily sedated, immobilized, and often socially isolated. In this setting, generalized MT might help establish a better environment, allowing to keep patients more awake during the day, cognitively engaged, and physically active, facilitating the patient’s autonomy and ability to express unmet physical, emotional, and spiritual needs [44].

The complexity of the ICU environment, along with the lack of point of reference of the patient can represent a horrifying situation. Moreover, encephalopathy can make it difficult for patients to outsource the multiple painful stimuli they feel, constricting them and their family members to surrender all control [45].

In all this perceived chaos, some patients with a critical illness may lose their humanity. This can take many forms, including the loss of personal identity, control, respect, privacy, and support systems, and is referred to as dehumanization: it consists of treating someone as an “object” rather than a “person” and is often associated with failure to respect dignity [46]. The reorienting effect of music, together with the pleasure of listening and recollections of the previous everyday life could help build a human-centered care ICU model [47].

### Limitations of the study

The investigation described here has several important limitations. First, it is hard to consider—in each survey—the main differences, such as the pathology, the operators in service, the number of interruptions, the volume of music, and noise pollution. Second, for comatose or sedated patients, it is impossible to establish—if they have recent hearing loss—whether the standard volume used is appropriate for them. Third, it is impossible, even with the music therapist’s best efforts, to satisfy all the patients’ musical tastes, especially in the personalized MT. Moreover, in the present study, only seven daily playlists for generalized MT are used; patients staying in the ICU for longer than 7 days hear the same songs each week. So far, only a few ICU staff members have complained about this, possibly because of the amount of music played (12.5 h per day). Fourth, the two treatment protocols in the design of our study differ in numerous aspects (intervention duration, device used for music listening, and method of track selection), which could independently affect the results, acting as confounding factors. Fifth, some technical problems have been reported, like disconnection of the loudspeakers’ wi-fi or headphones that come off.

Lastly, we have yet to report effective MT generalizability, making this an interesting challenge. The literature indicates that music can have similar effects in different people, not only in terms of personal tastes but also through vital parameters such as heartbeat [26] or feeling of relaxation [48]. Furthermore, the emotions the music conveys are largely generalizable: if a song sounds happy, most listeners will respond with neurophysiological activation relating to joy and will experience pleasure [49]. This supports the hypothesis that music can evoke similar emotions and experiences, allowing us to hope that specific tracks will reduce stress, anxiety, and pain patients may experience in the ICU.

## Conclusion

The “Generalized music therapy for the critically ill” study is a scientific investigation aiming to transpose the advantages of MT into ICU daily practice, evaluating the effectiveness and feasibility of a generalized approach. Then, if it proves useful in reducing the need for neuroactive drugs, this MT protocol could be offered to other Italian ICUs.

Moreover, if the results are encouraging, the rules used to prepare the playlists could be used as a basis for other lists in different places, based on local culture and preferences. This study could also offer some inspiration to design ICU devices (like mechanical ventilators) with built-in music speakers or to establish an Internet-radio channel to make specific MT available for ICU patients. At this stage, we found that generalized MT seemed feasible in the ICU and was viewed positively by staff, critically ill patients, and their families.

## Trial status

The current study protocol version is no. 2, January 9, 2017. The study has already started enrolment, but the completion was delayed due to the severe impact that the CoViD-19 pandemic spike caused on the organization of our research center; we expect to finalize the procedures by March 31, 2024.

### Supplementary Information

Additional file 1: 1. General criteria for the selection of music tracks. 2. Specific criteria for time slots. 3. Technical features of generalized MT. 4. Italian references used by the music therapist to compose the daily playlists of generalized MT.5. Music Assessment Tool (MAT)—Italian Version. 6. Italian Data Collection Sheets. 7. Web link for the full list of all tracks used in the generalized MT.

## Data Availability

The principal investigator and the senior investigator (MU and GM) will have access to the final trial dataset.
