# Supplementary material for: Generalized music therapy to reduce neuroactive drug needs in critically ill patients. Study protocol for a randomized trial
Source: Trials. 2024 Jun 12;25:379. doi: 10.1186/s13063-024-08220-8 (PMC11170779; doi:10.1186/s13063-024-08220-8)
Supplement: Supplementary file 1 — Additional file 1: 1. General criteria for the selection of music tracks. 2. Specific criteria for time slots. 3. Technical features of generalized MT. 4. Italian references used by the music therapist to compose the daily playlists of generalized MT.5. Music Assessment Tool (MAT)—Italian Version. 6. Italian Data Collection Sheets. 7. Web link for the full list of all tracks used in the generalized MT. [file 13063_2024_8220_MOESM1_ESM.docx]

**Generalized music therapy to reduce neuroactive drug needs in critically ill patients. Study protocol for a randomized trial**

**Running title:** Generalized music therapy for critically ill patients

Giovanni Mistraletti^1,2^, Anna Solinas^3^, Silvia Del Negro^4,5^, Carlotta Moreschi^4^, Stefano Terzoni^4^, Paolo Ferrara^4^, Katerina Negri^1^, Paolo Formenti^6^, Angelo Formenti^7^, Michele Umbrello^2^

^1^ Dipartimento di Fisiopatologia Medico-chirurgica e dei Trapianti, Università degli Studi di Milano.

^2^ SC Rianimazione e Anestesia, Ospedale Nuovo di Legnano, ASST Ovest Milanese, Milano.

^3^ Dipartimento di Salute Mentale, AUSL Piacenza.

^4^ Dipartimento di Scienze della Salute, Università degli Studi di Milano.

^5^ Servizio di Psicologia Clinica, Ospedale San Paolo – Polo Universitario, ASST Santi Paolo e Carlo, Milano.

^6^ UOC Anestesia e Rianimazione I, Ospedale San Paolo – Polo Universitario, ASST Santi Paolo e Carlo, Milano.

^7^ Centro Sperimentale Regionale della Voce e della Deglutizione “E. De Amicis”, Milano.

**ELECTRONIC SUPPLEMENTARY MATERIAL**

1 – General criteria for musical tracks choice

2 – Specific criteria for time slots

3 – Technical features of generalized MT

4 – Italian references used by the music therapist to build the daily playlists of generalized MT

5 – Music Assessment Tool (MAT) – Italian Version

6 – Italian Data Collection Sheets

7 – Web link for the full list of all tracks used in the generalized MT

**1 – General criteria for musical tracks choice**

1. Non-aggressiveness, straight-forwardness, and regularity in the musical parameters (except for bands 4 and 6, where a considerable increase in energy is required, see table 1).
   1. Dynamics should be mostly constant, without sudden peaks (such as timpani, cymbals, brassens); where these were occasionally present, they were adjusted with compression of the dynamic range. Anticipated and gradual increases and decreases in dynamics were also introduced.
   2. Predominantly regular-agogic pattern, without abrupt interruptions or accelerations.
   3. Timbre indifferently isolated or mixed, but not pungent, harsh or gloomy (unless muffled by the presence of clear and soft opposite stamps; allowed in bands 4 and 6). Vocal and instrumental emissions mostly soft and sweet; at times, it could be quite sharp, but not rough or hard (except for bands 4 and 6).
   4. Predominantly medium and acute frequencies. Serious tones not isolated or only for short periods (allowed in band 5 and partially in band 7).
   5. Simple and easily predictable melodic lines.
   6. Complete and regular phraseology in the engravings, sentences and periods.
   7. Simple and regular rhythmic structure, mainly free of syncopation and accentuation (excluding bands 4 and 6).
   8. Formal structure present and perceptibly recognizable.
   9. Development of the tracks should be either tied to specific patterns, detached or mixed.
   10. Predominantly higher shades and tonalities- lower shades prevailing only in band 8.
   11. Tonal harmonies; non-tonal, modal or dodecaphonic harmonies.
   12. Competition of all musical parameters to the expression of mainly positive, serene and reassuring emotions and atmospheres.
2. Music genres were as varied and popular as possible. The unusual music genres (examples: Gregorian chant, Neapolitan neo-melodic, free jazz, ...), heavy metal, hard rock, and techno/disco genres, were excluded
3. A variety of styles and eras, also within genres, was selected. Non-affirmed or experimental styles were excluded
4. Preference for high-quality engravings and executions was a relevant cornerstone.
5. The repertoire is taken from the Western cultural heritage, generally after the year 1600.
6. Each choice was made by analyzing the phono-symbolic meaning of the musical parameters while being aware that it remains impossible to predict which synesthetic associations may occur in each patient, being these specific, unique and the most varied for each individual.
7. The selection of songs was determined by thirty years of experience in music therapy in the psychiatric field and dozens of interviews with people of various age groups.

**2 – Specific criteria for time slots**

**BAND 1** – Sensory revival – from 07:00 to 08:00

| Frequency | Medium-acute |
| --- | --- |
| Dynamics | Low, progressively increasing |
| Agogic pattern | Slow or very slow in progressive acceleration |
| Timber qualities - Emission | Light and soft timbers, in their specific quality and emission (female and children's voices - strings and horns) |
| Melodic line | Simple |
| Tonality | Predominantly major |
| Phraseology | Simple and complete, medium length |
| Formal structure | Simple and regular |
| Rhythmic structure | Beat absent or in the background, progressively more evident. Regular. |
| Articulation | Moslty linear in the first tracks. Progressive introduction of *staccato* and *pizzicato* |
| Text | Indifferently present (often in a foreign language), or absent |
| Degree of notoriety | Predominantly medium-low |
| Phonosymbolism  (in brackets some probable synesthesias) | A slow agogic pattern, with low dynamics progressively increasing to reproduce the slowdown of vital functions during sleep and their gradual acceleration and intensification after awakening. The absence of a rhythmic section highlighted in the initial passages reflects the absence of perception of the heartbeat when it is weak and slowed upon awakening. The clear timbres recall daylight. Soft melodies and emissions evoke a gentle awakening (such as caresses and whispering). The tied framework recalls a relaxed and elongated movement, while a detached and punctiform one, suggests a small and light contact on a small surface. The stronger tonalities suggest a positive mood. The medium-high frequencies suggest an elevation (of the gaze, of the body, ...). The absence of text, or presence of them in a foreign language, as well as the low-medium notoriety, allow attention to be drawn to the music and its parameters, which are, in this case, more effective than the text for achieving the prefixed objective. |

**BAND 2** – Encouraging imagination – from 08:00 to 09:00

| Frequency | Medium-acute |
| --- | --- |
| Dynamics | medium |
| Agogic pattern | medium |
| Timber qualities - Emission | Soft or clean timbres, soft or clean emission, great variety of coloristic shades |
| Melodic line | Simple and predictable |
| Tonality | Predominantly major |
| Phraseology | Medium-long |
| Formal structure | A large scheme (*sonata*), or free of any particular one |
| Rhythmic structure | Simple, with perceptible beating possibilities, but not heavy and constant |
| Articulation | Combination of *legato* and *staccato* techniques |
| Text | Indifferently present (often in a foreign language), or absent |
| Degree of notoriety | Medium-high |
| Phonosymbolism  (in brackets some probable synesthesias) | The mid-acute melodic line focuses on high areas of himself. The long and medium-speed phrases prolong and let the imagination flow, in a course parallel to the colors proposed by the timbre variations of the full orchestra.  The genre "film music" and "symphonic", mainly used in this band, can evoke memories of scenes observed or lived. The perceptible beat adds realism to the imagination. The higher tone gives a positive connotation to self-shaped images. The absence of text, or the use of a foreign language, allows to direct attention to the music and its parameters, which are, in this case, more effective in achieving the goal. The medium-high notoriety of the pieces, often linked to film scenes, or the descriptiveness of the symphonic pieces, allows visual memories to emerge. |

**BAND 3** – Muscle reactivation – from 09:00 to 11:00

| Frequency | Medium or mixed |
| --- | --- |
| Dynamics | Constant |
| Agogic pattern | medium |
| Timber qualities - Emission | Soft or clean emission. Coloristic simplicity (two/three timbres, usually combined in breathes/voice plus chordophones and percussion) |
| Melodic line | Ascending and descending in regular alternation |
| Tonality | Major or minor |
| Phraseology | Short, engraved between the music lines |
| Formal structure | Simple and regular |
| Rhythmic structure | Regular with a clearly perceptible beat |
| Articulation | Combination of *legato* and *staccato* |
| Text | Mainly absent or present in foreign language (dialect or ancient talk) |
| Degree of notoriety | Predominantly low |
| Phono symbolism (in brackets some probable synesthesia) | The mid-acute melodic line focuses on high areas of itself. The long and medium-speed phrases prolong and let the imagination flow, keeping constant with the colors proposed by the timbre variations of the full orchestra.  The medium-slow time reproduces the real speed of mobilization exercises during physiotherapy.  The melodic line, ascending and descending in regular alternation, evokes a movement of raising and lowering, of contraction and relaxation (of limbs and muscles).  The combined soft and clear tones suggest relaxation together with maintenance of the muscle tone.  The mixed joint, bound and detached, evokes deep breathing, a sequence of movements as a whole, resulting in a perception of burning (tension) and stretching (relaxation), and so also the mobilization of the various parts of the body.  The absence of text, or the use of a foreign language, as well as the low level of notoriety, make it possible to direct attention to music and its parameters, which are, in this case, more effective than the text for achieving the goal. |

**BAND 4 and 6** – Daily life – from 11:00 to 12:00 and from 15:00 to 17:00

| Frequency | Medium or mixed |
| --- | --- |
| Dynamics | Supported |
| Agogica | Medium-rapid |
| Timber qualities - Emission | Intense colours and emissions, even forced and rough. |
| Melodic line | Short and simple |
| Tonality | Predominantly major, not excluding the minor |
| Phraseology | Short and clear |
| Formal structure | Simple, regular and predictable |
| Rhythmic structure | Simple and with very obvious beats, with a possibility for accents even in syncope |
| Articulation | Mixed / blended |
| Text | Independently present or absent in Italian or foreign/antique language |
| Degree of notoriety | medium |
| Phonosymbolism  (in brackets some probable synesthesias) | The sustained dynamics, rapid timing and rhythmic accents suggest great concentration of energy and evoke positive emotions (courage, strength, determination...).  The text, if present, can contribute, in second place to the vocal emission, to give energy and strength to the pieces.  Notoriety is possible but not necessary to achieve the desired purpose in this band. |

**BAND 5 and 7** – Memories – from 12:00 to 15:00 and from 17:00 to 19:00

| Frequency | Mixed (possibility of holding high notes) |
| --- | --- |
| Dynamics | Average, with the possibility of expressive increases and decreases |
| Agogic pattern | Mixed / blended |
| Timber qualities - Emission | The most different |
| Melodic line | Searching to express one’s own highlights, with melodic climaxes and large openings |
| Tonality | Majors and minors |
| Phraseology | Medium and well perceivable |
| Formal structure | Simple and regular |
| Rhythmic structure | Regular |
| Articulation | Mixed / blended |
| Text | Mainly in Italian, dialect or foreign language |
| Degree of notoriety | High |
| Phonosymbolism  (in brackets some probable synesthesias) | Since these are well-known or famous pieces, the emotional component is mainly linked to individual memories and experiences or the mimesis of feelings and emotions through isomorphism.  The notoriety of the pieces and the presence of the text can contribute to the emotional memory or to convey the expression of pleasant emotions and feelings. |

**BAND 8** – Consolatory empathy - from 19:00 to 21:00

| Frequency | Mixed / blended |
| --- | --- |
| Dynamics | Medium-low |
| Agogic pattern | Medium-slow, slowing down |
| Timber qualities - Emission | Mixed, soft and sweet |
| Melodic line | Simple and highlighted, tendency to downward motion |
| Tonality | Predominantly minor |
| Phraseology | Simple and medium length |
| Formal structure | Simple and regular |
| Rhythmic structure | Very simple and regular, often cradling and cadenced |
| Articulation | Mixed / blended |
| Text | Independently present or absent in Italian or foreign, ancient and dialect languages |
| Degree of notoriety | medium |
| Phonosymbolism (in brackets some probable synesthesias) | The minor tones give a melanchonic, self-absorbed and intimate connotation suggesting a tuning with the patients' suffering.  The lulling and cadenced rhythms allow to perceive at the same time a sense of care and containment of pain.  The gradual slowing down of the pieces favors a stepwise relaxation.  The presence of a text is not as fundamental as the quality of the vocal emission for the achievement of the prefixed purpose in this band.  The notoriety of the songs is not as fundamental to the performance as the musical parameters indicated above. |

**BAND 9** – Sleep-inducing relaxation - from 21:00 to 22:00

| Frequency | Mixed / blended |
| --- | --- |
| Dynamics | Contained |
| Agogic pattern | medium |
| Timber qualities - Emission | Mixed but reduced to a few colors, soft or clean emissions |
| Melodic line | Circular movement |
| Tonality | Major or minor |
| Phraseology | Short / brief |
| Formal structure | free |
| Rhythmic structure | Repetitive with highlighted beats |
| Articulation | Mixed / blended |
| Text | absent |
| Degree of notoriety | low |
| Phonosymbolism  (in brackets some probable synesthesias) | The use of constant rhythms mimic a regular heartbeat and their predictability relaxes the thought. These rhythms, together with the circular repetition of the melodies in the foreground, and with slight variations in the accompaniment and the timbre in the background, reproduce the hypnotic mechanisms that focus attention on an element repeatedly proposed, neutralizing the emotions. The micro variations on the background make the melodic and rhythmic re-proposal as not recurring.  These are pieces of recent compositions in which the circular melody together with the repetitive rhythm produces the effect of relaxation. |

**Total music production: 50' every hour x 15 hours/day = 12,5 hours/24**

**NOTE:**

Since the selection of the pieces was made on already published material (existing works, without ad-hoc compositions), there is the possibility that the ideal qualities of the musical pieces may not be present in part or may not fully conform to the above-mentioned characteristics. Moreover, each piece may possess characteristics typical of two or more bands, but its inclusion in only one of these has been determined by the predominant characteristics.

**3 – Technical features of the playlist’s tracks**

1. The songs are compressed in MP3 format (Moving Picture Expert Group - 1/2 Audio Layer 3) at a transmission speed of 128 kbps.
2. The pieces have been selected and divided into nine time bands, each containing a different type of music intended to fulfill different purposes throughout the day.
3. Of some tracks, only the section corresponding to the chosen type has been extrapolated, eliminating the parts that do not conform to the types indicated above.
4. In the songs recorded live, the applause, the presentations and everything that could generate confusion concerning the time band and category in which the song had been inserted have been removed or blurred.
5. In some tracks it was chosen to change the volume at the beginning and/or end of the song, to make them fade in or out.
6. The tracks are clipping-free.
7. The tracks have no background noise (if not intentionally inserted in the track by the author such as water or wind noise) except for the original reproductions of the 40s and 60s that sometimes report the noise of old playback media (LP discs, poor quality radio or television recordings, etc.).
8. The tracks have all been normalized to avoid volume changes in sequential execution and sound waves positioned below (i.e. at least -10%) the distortion threshold. In some cases, the normalization has also been done separately on the two stereo channels in order to decrease the over accentuated stereo effect.
9. The acoustic intensity (i.e. the volume, described as the ratio between sound intensity and threshold of audibility) of the music perceived by the patient's ear during "generalized music therapy" treatment should be at least 5 dB higher than the average intensity of the background noise in the ward, so that it is possible to follow the music and to make it easily recognizable from the background noise.
10. Some tracks have had a compression of the dynamic range (with a ratio equal to 2:1, while in very few cases to 4:1) intending to implement the signals that arrive weak at the input without intervening on those that already have a high level (upward compression) to make it possible to listen to them in low volume reproductions.

**4 – Italian references for MT**

Books used by the music therapist to build the daily playlists of generalized MT

AA.VV., Musicoterapia ad indirizzo fenomenologico, Urbania, Progetti Sonori, 2006.

Benenzon R., Manuale di musicoterapia, Roma, Borla, 1983.

Dropsy J., Vivere nel proprio corpo. Espressione corporea e rapporti umani, Ottaviano, Milano, 1988.

Gaita D., Il pensiero del cuore. Musica simbolo inconscio, Milano, Bompiani, 2000.

Giordani B., Psicoterapia umanistica da Rogers a Carkhuff. La terapia centrata sulla persona, Cittadella, Assisi, 1988.

Guerra Lisi S., Come non spezzare il filo, Roma, Borla, 1990.

Imberty M, Suoni emozioni significati, per una semantica psicologica della musica, Bologna, CLUEB, 1986.

Lecourt E., La musicoterapia, Assisi, Cittadella, 1999.

Lorenzetti L.M., Antonietti A., a cura di, Processi cognitivi in musica, Milano, Franco Angeli, 1986.

Sacks O., Musicofilia, Milano, Adeplhi, 2010.

Postacchini P., a cura di, Emozioni e musicoterapia, Assisi, PCC, 1997.

Postacchini P., Ricciotti A., Borghesi M., Lineamenti di musicoterapia, Urbino, Carocci, 1997.

Salvadori C., Salvadori S., a cura di, Musica e salute. L’azione del musicista nei contesti di cura, Torino, EDT, 2006.

Stefani G., Competenza musicale e cultura della pace, Bologna, CLUEB, 1985.

Stefani G., Ferrari F., a cura di, La Psicologia della Musica in Europa e in Italia, Bologna, CLUEB, 1986.

**5 – Music Assessment Tool (MAT)**

Italian Version


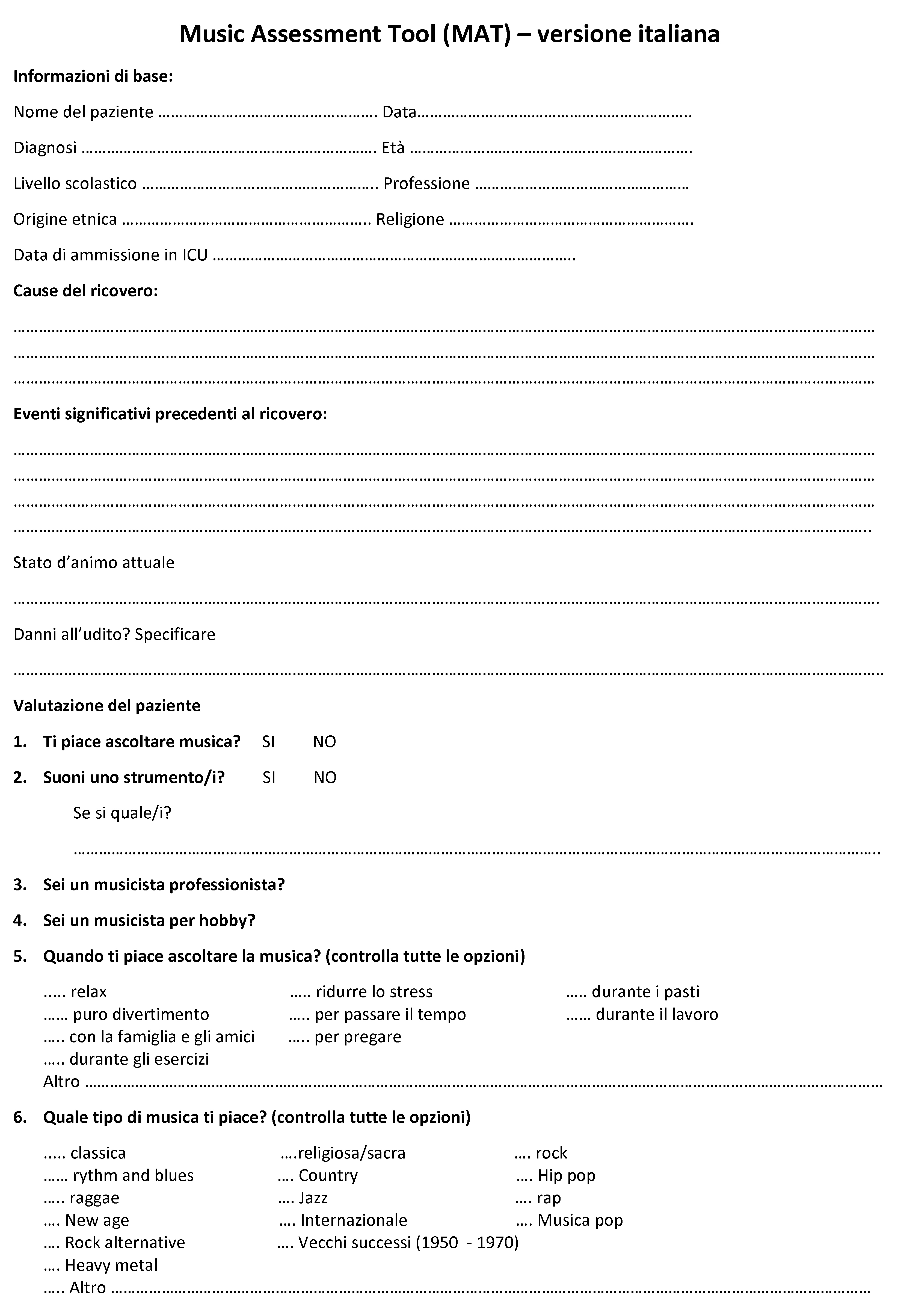


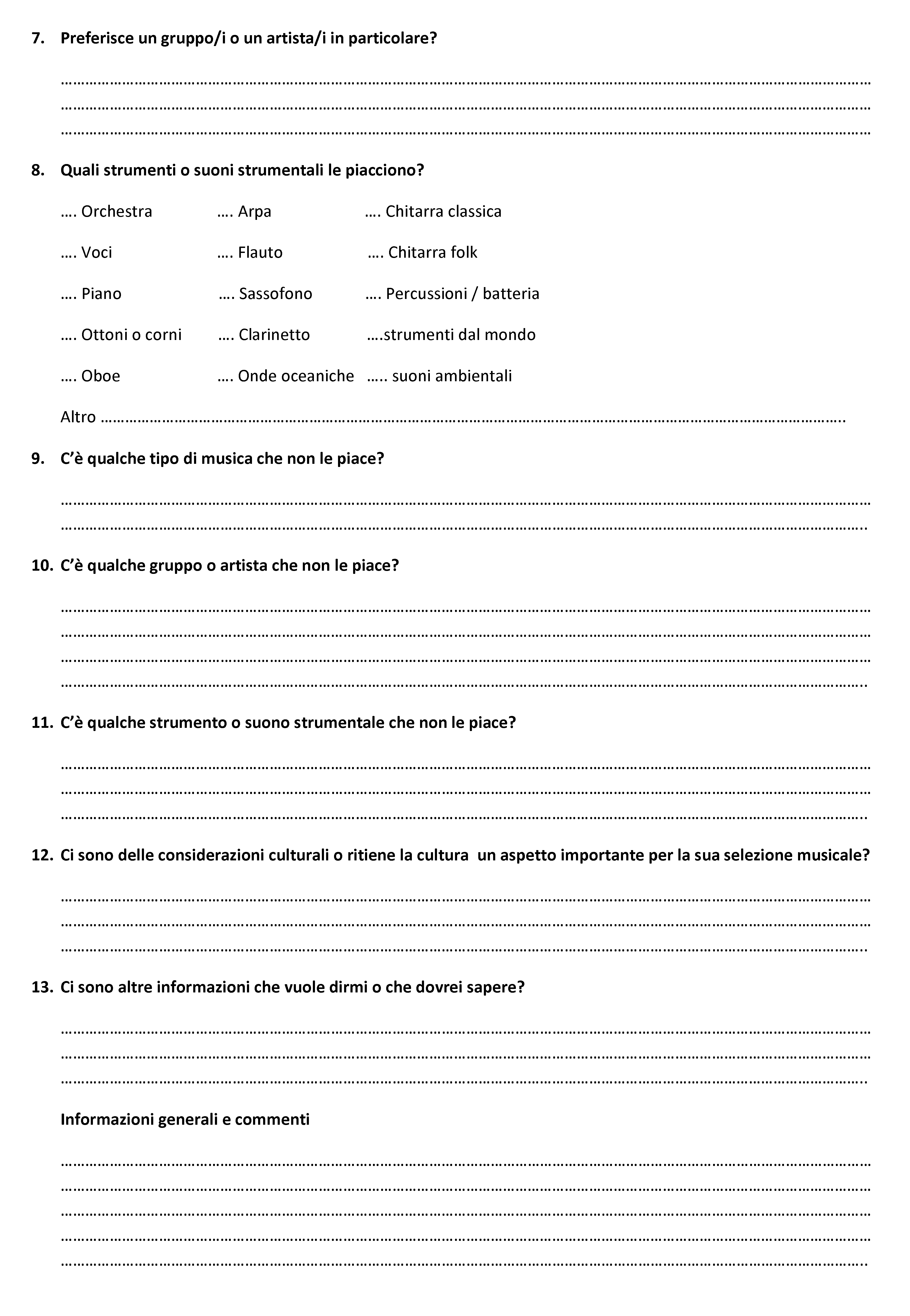


**6 – Italian Data Collection Sheets**


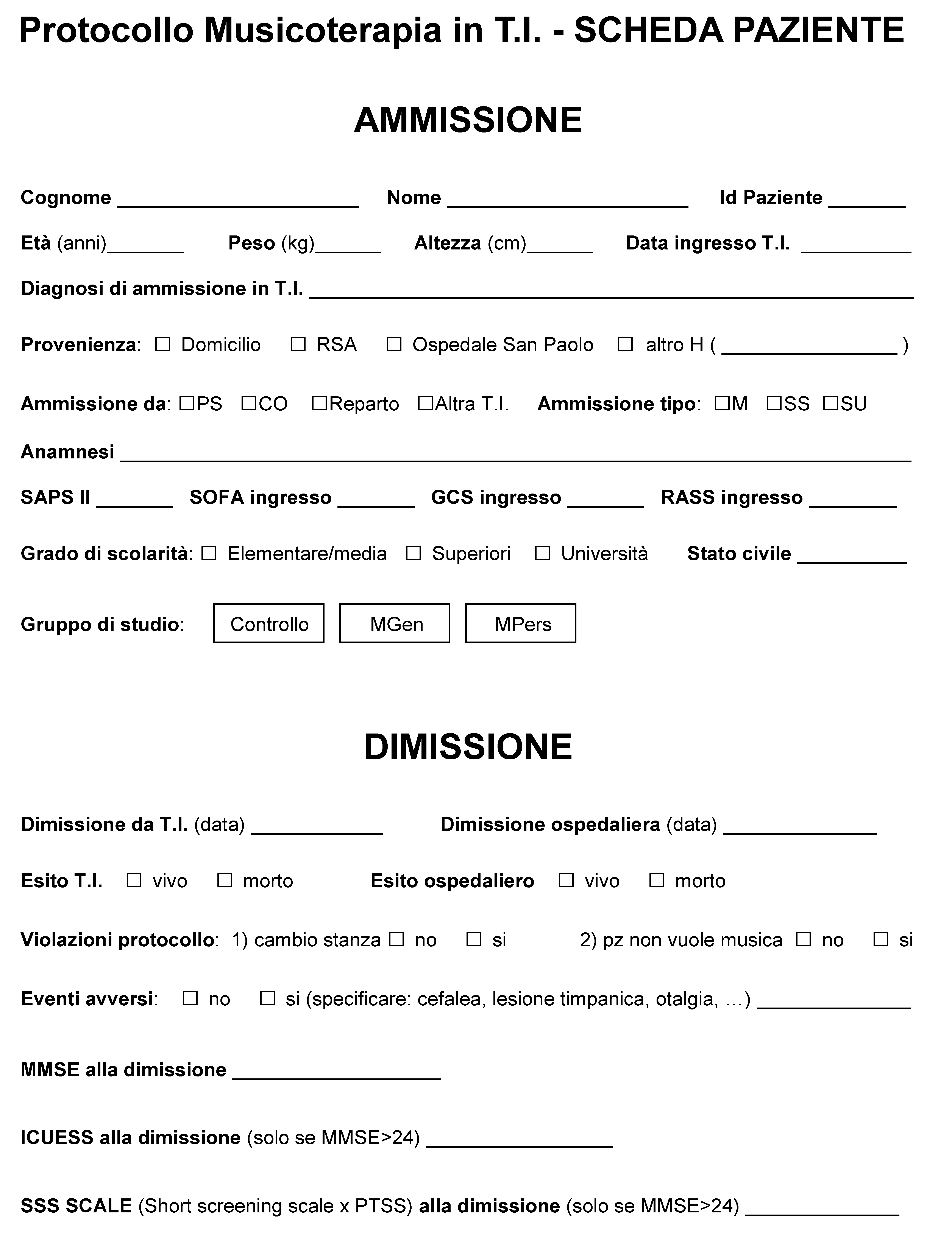


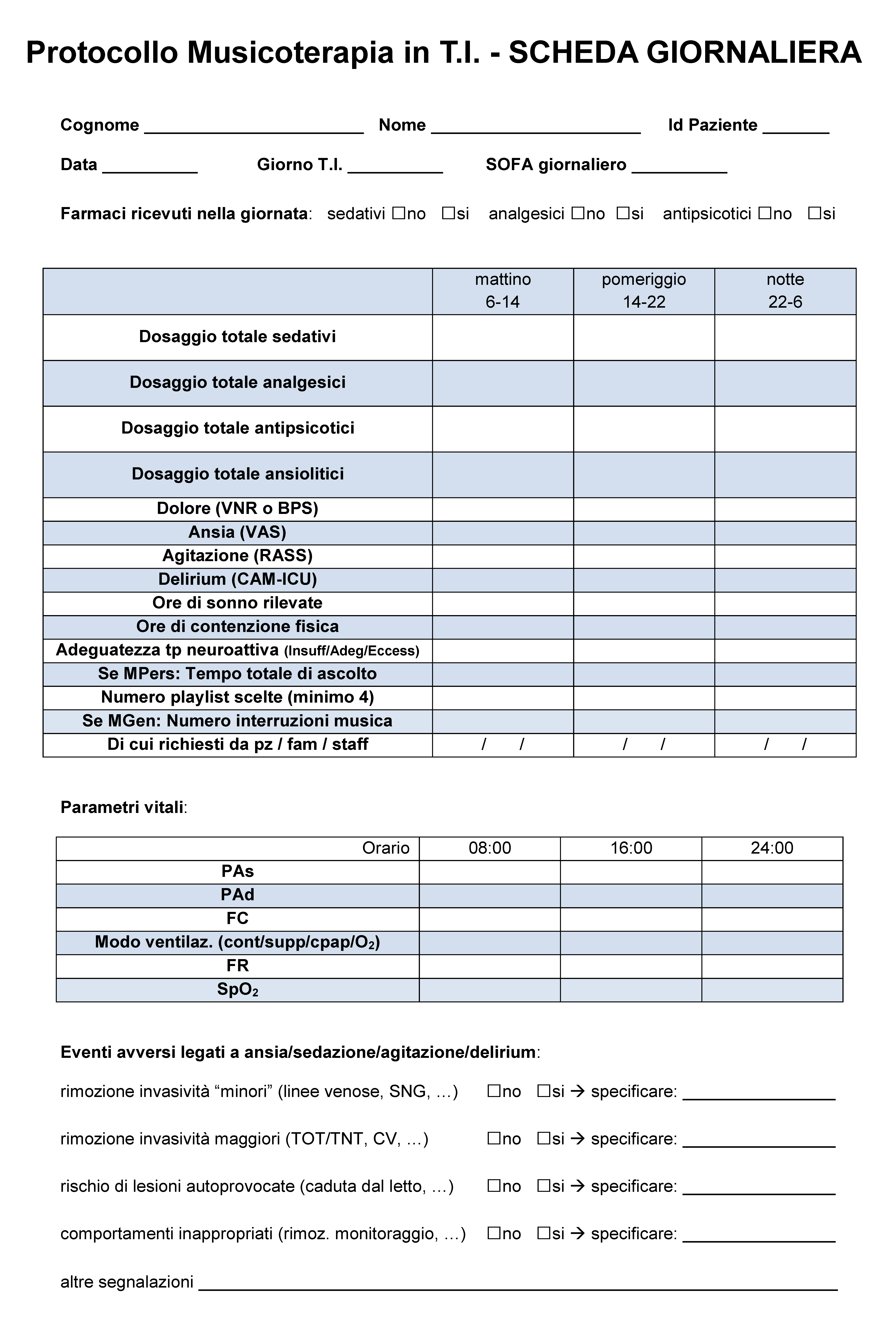


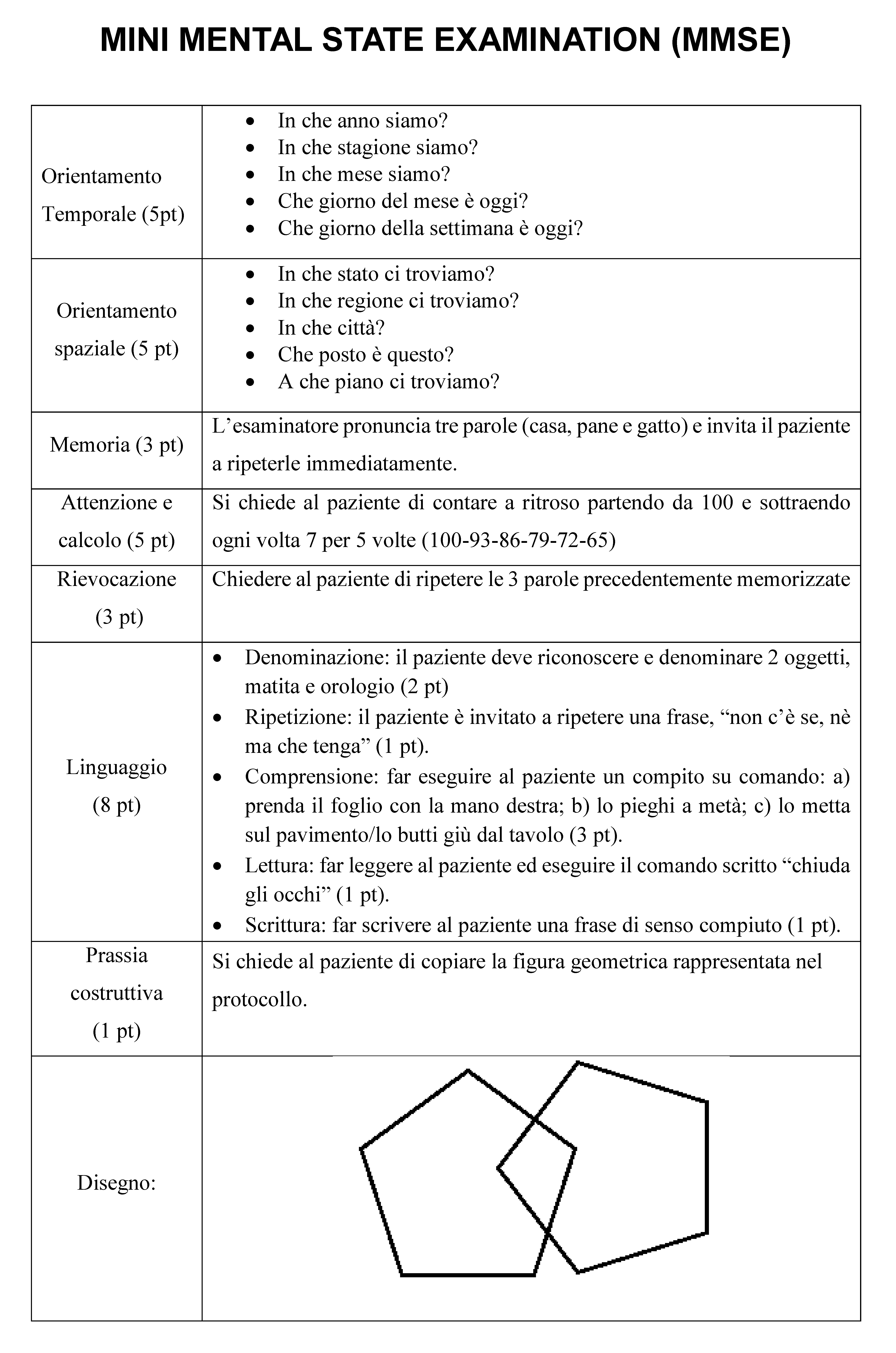


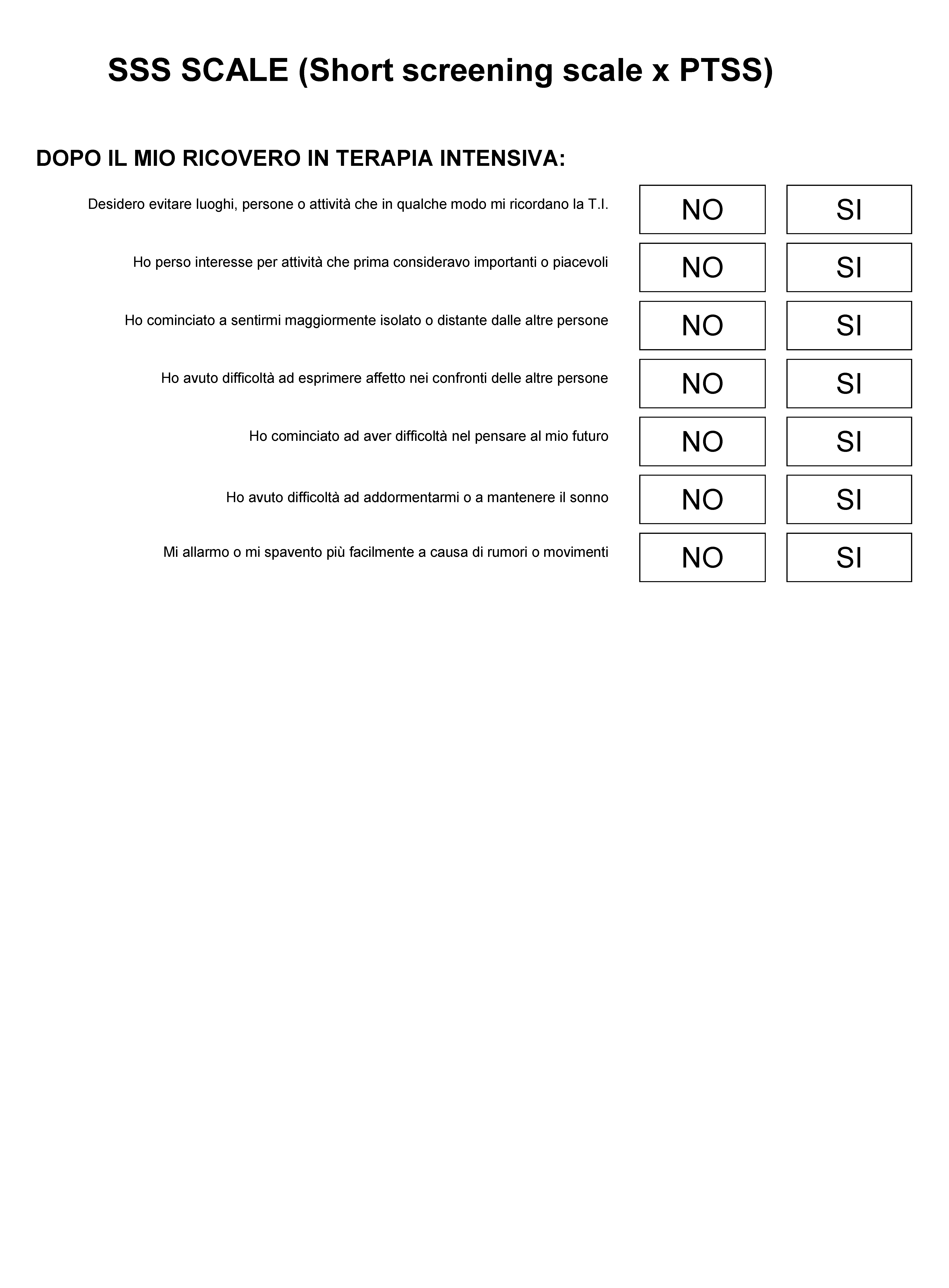


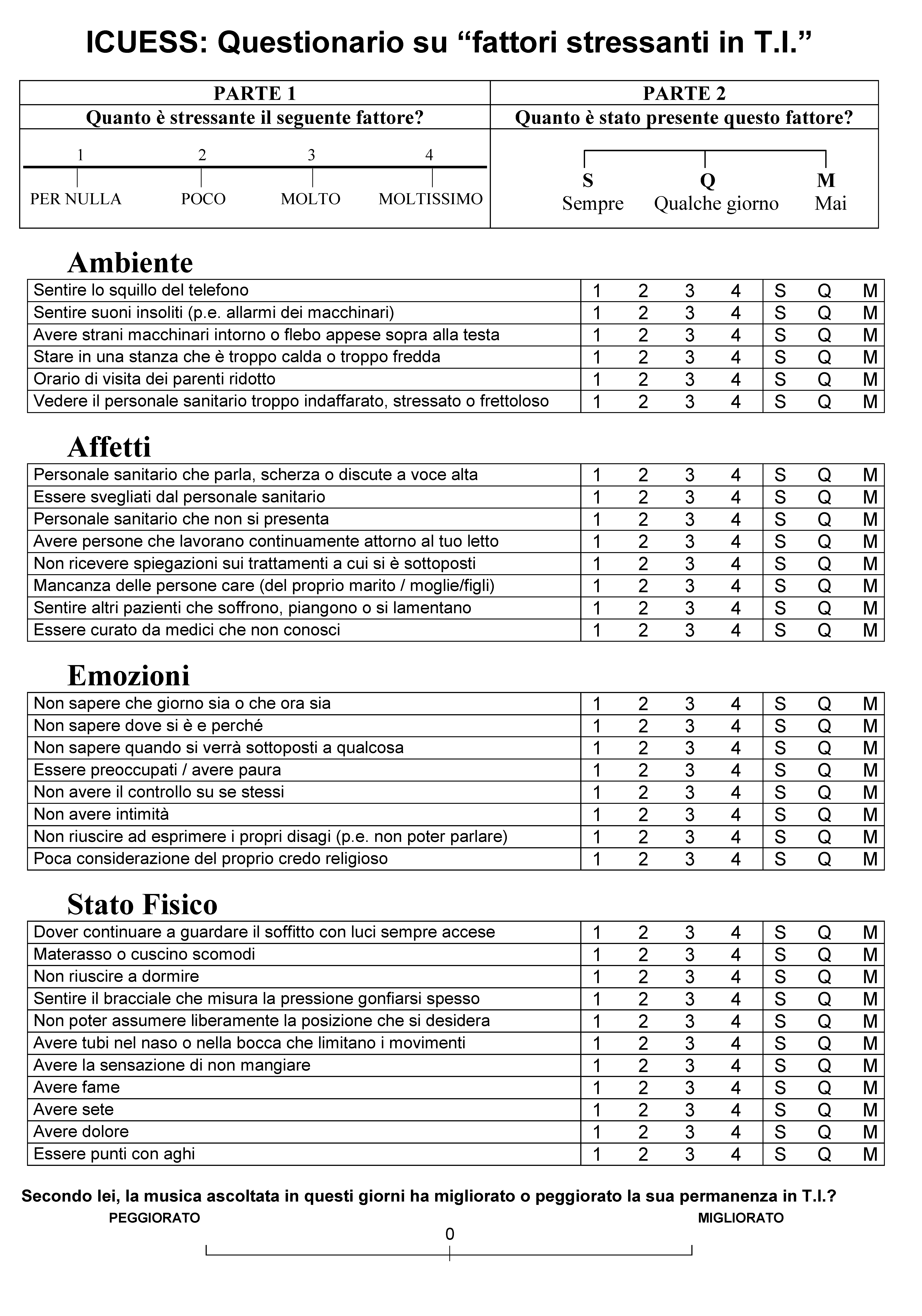


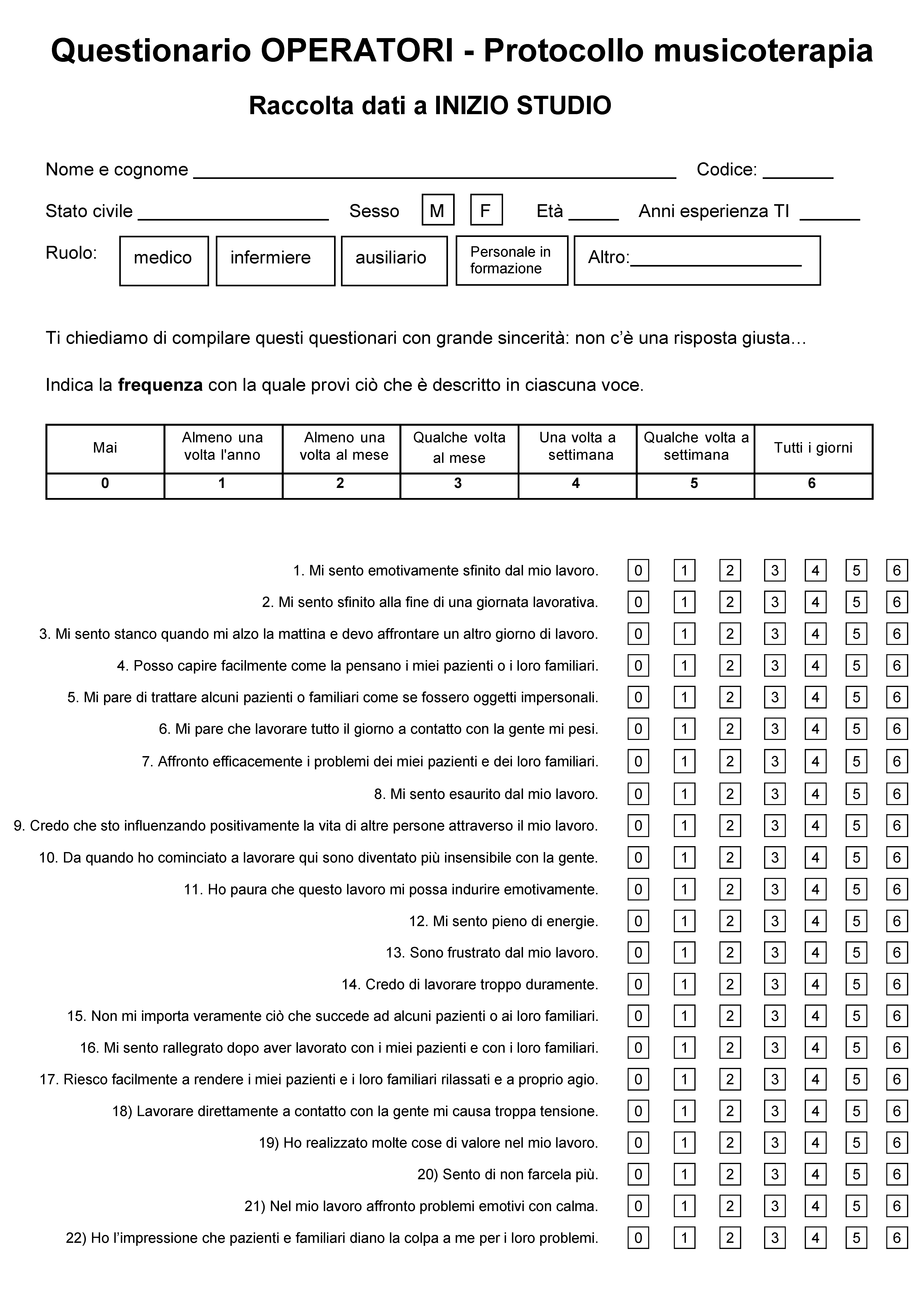


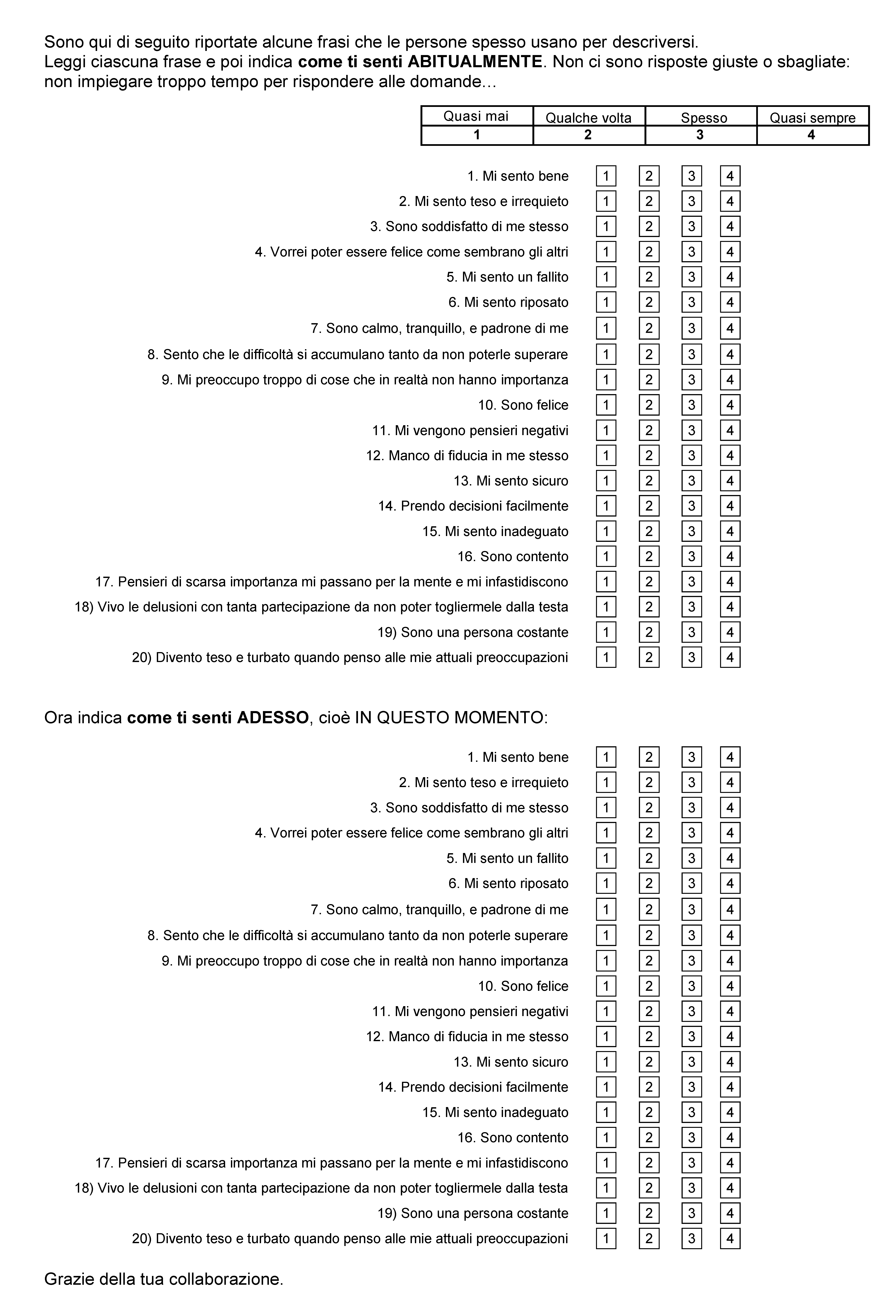


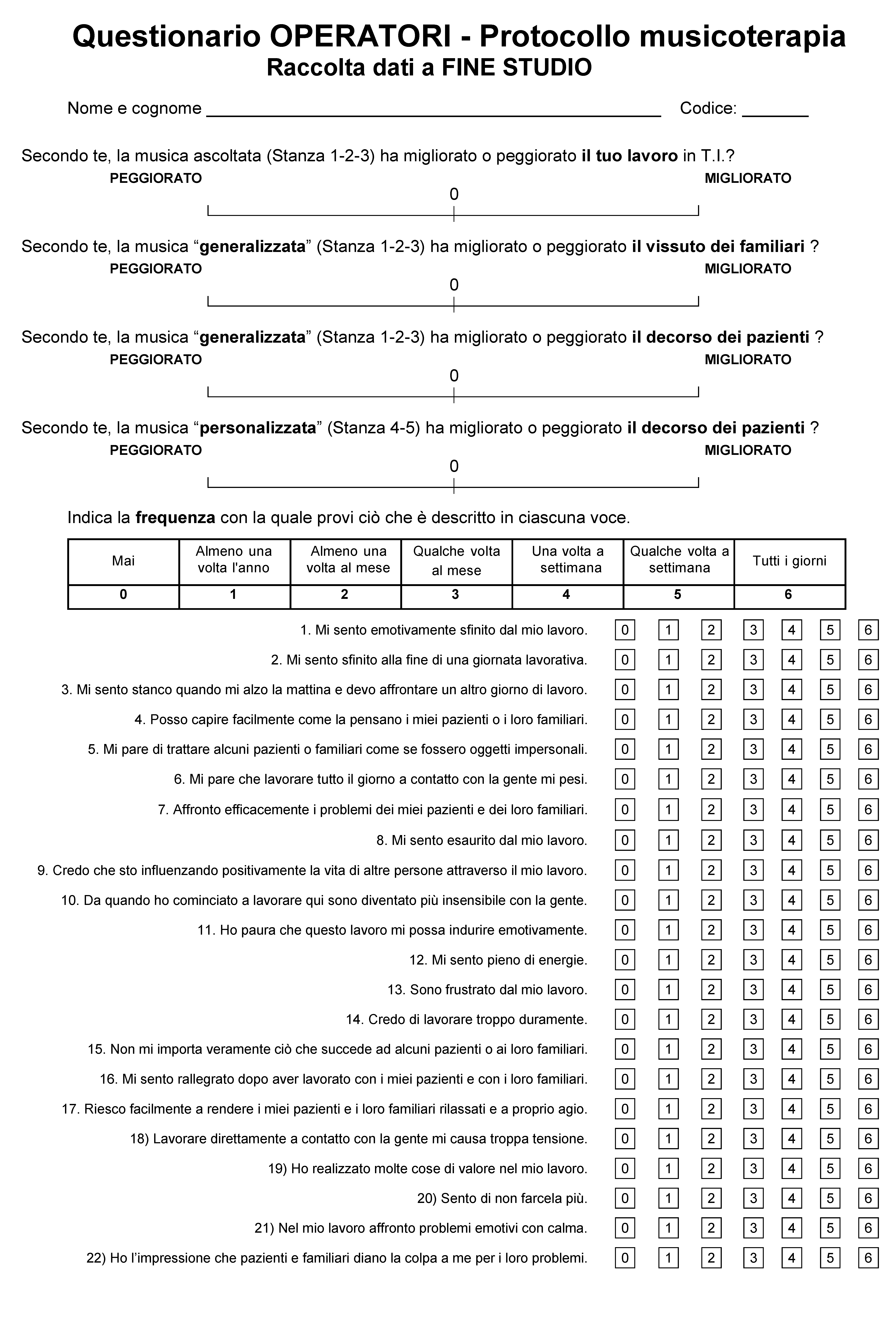


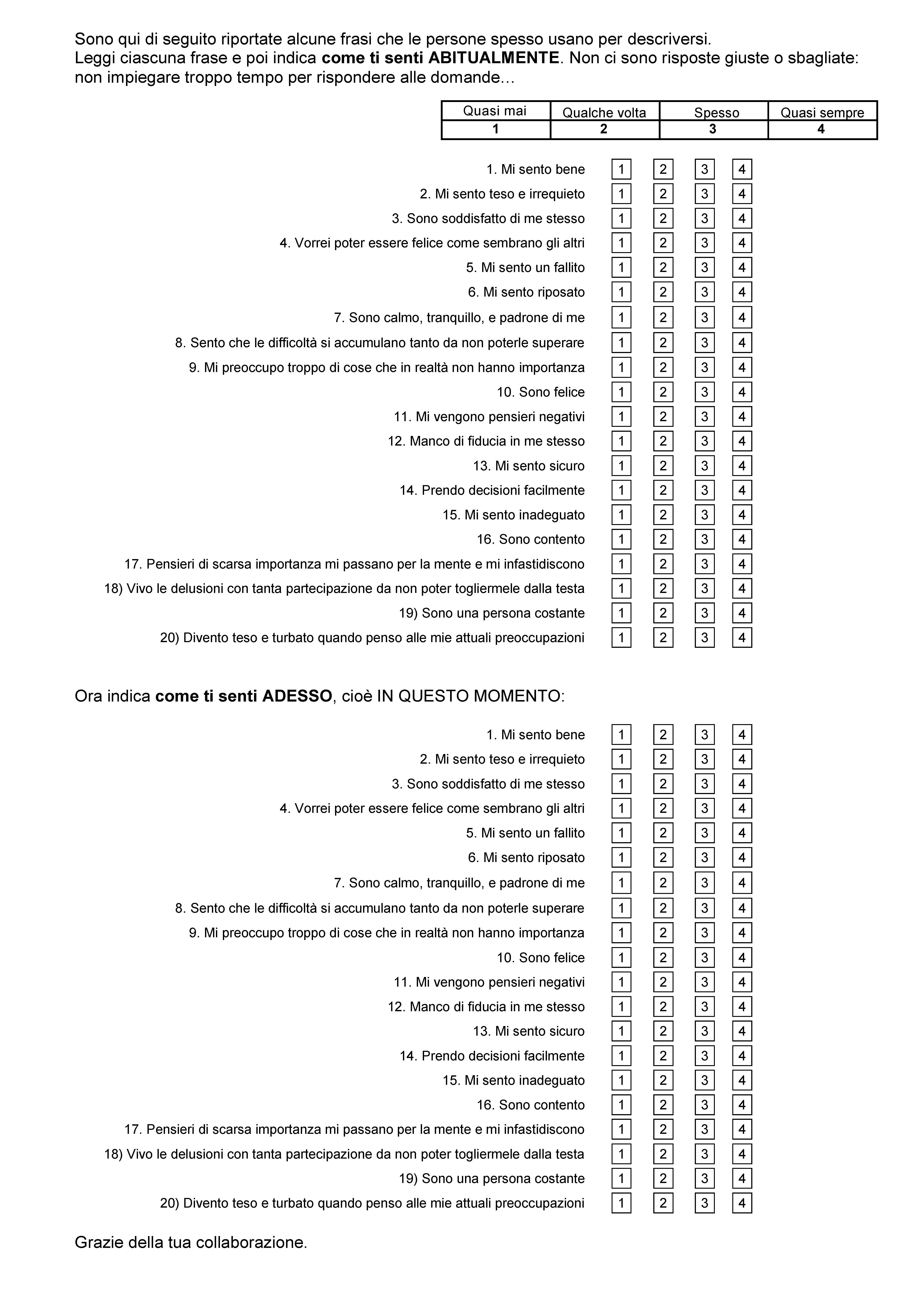


**7 – Full list of generalized MT tracks.**

At this web link is freely available a .xlsx file describing the full list of all tracks used in the generalized MT:

[ElencoBraniMusicoTpICU-22feb18](https://www.dropbox.com/s/nf6yt5yh4e2hwst/ElencoBraniMusicoTpICU-22feb18.xlsx?dl=0)
